# Lithium: more than a mood stabilizier

**DOI:** 10.1192/j.eurpsy.2022.1027

**Published:** 2022-09-01

**Authors:** A. Fraga, B. Mesquita, J. Facucho-Oliveira, M. Albuquerque, P. Espada-Santos, P. Cintra, S. Paulino, A. Moutinho

**Affiliations:** Hospital de Cascais, Psychiatry, Alcabideche, Portugal

**Keywords:** bipolar disorder, Neuromodulatory, Lithium, White matter

## Abstract

**Introduction:**

Bipolar disorder (BD) is characterized by episodic changes in affect, motivation, cognition and behavior. This severe mental disorder has a prevalence of at least 1% and a high heritability of 60%-80%. The pathophysiology is still poorly understood but evidence indicate that the disorder relates to disturbances in front-limbic networks relevant for emotion processing and regulation. New techniques have been used to study BD and showed aberrante white matter (WM) microstructure in the corpus callosum and from-limbic pathways. However, lithium, a mood stabilizier, it looks like has celular and neuromodulatory effects.

**Objectives:**

The authors elaborate a narrative literature review to identify the existing clinical evidence of lithium’s effect on the WM from BD patients.

**Methods:**

Pubmed databased searched using the therms “bipolar disorder”, “white matter” and “lithium”.

**Results:**

Lithium is a bipolar medication that confers treatment and long-term prophylaxis and been reported as having neuroprotective effects.

Studies that used new techniques such diffusion tensor imaging measures to assess white matter integrity reported a positive effect of lithium on the integrity of WM of BD patients and suggest that response to lithium treatment in BD patients is associated with normalization of WM microstructure in regions associated with emotion processing.

**Conclusions:**

Lithium appears to positively influence the evolution of the white matter abnormalities described in BD patients however further investigation is required to strongly reinforce this potential and safety.

**Disclosure:**

No significant relationships.

